# A Wizard-of-Oz Shuttle as an Experimental Platform for Research on Future Automated Public Transport

**DOI:** 10.3390/s26206578

**Published:** 2026-10-16

**Authors:** Khazar Dargahi Nobari, Tobias Heinrich Motal, Sven Gravemeier, Daryna Sytnychenko, Torsten Bertram

**Affiliations:** Institute of Control Theory and Systems Engineering, TU Dortmund University, Otto-Hahn-Str. 8, 44227 Dortmund, Germany; tobias.motal@tu-dortmund.de (T.H.M.); sven.gravemeier@tu-dortmund.de (S.G.); daryna.sytnychenko@tu-dortmund.de (D.S.); torsten.bertram@tu-dortmund.de (T.B.)

**Keywords:** automated driving, passenger monitoring, human-vehicle interaction, human factors, emulator

## Abstract

**Highlights:**

**What are the main findings?**

- Development of a Wizard-of-Oz (WoZ) concept for investigating human-machine interaction in automated shuttles operating in public transportation.
- Implementation of a WoZ-enabled shuttle and its laboratory-based analog twin as experimental platforms for human-subject studies.

**What are the implications of the main findings?**

- Integration of sensors and human-machine interfaces enables the investigation of passenger interactions and behavior in realistic and controlled experimental environments.
- Implementation of a software architecture for synchronized data acquisition enables systematic collection and analysis of multimodal sensor and interaction data.

**Abstract:**

Automated public shuttles have the potential to improve road safety, transport accessibility, and passenger experience. However, before such systems can be widely deployed, their sensing, interaction, monitoring, and assistance functions must be systematically designed and evaluated under realistic operating conditions. This paper introduces a platform for automated user-centred local mobility analysis (PAULA), a Wizard-of-Oz (WoZ) shuttle developed as a flexible experimental platform for research on future automated public transport. In the proposed approach, the dynamic driving task is performed by a concealed human driver, while the passenger monitoring, environmental perception, and interaction functions under investigation are implemented through the platform’s sensors, algorithms, and interfaces. The platform is implemented in a shuttle with nine passenger seats and integrates multiple sensors and human-machine interfaces (HMIs) for monitoring the vehicle interior and its surroundings, collecting behavioral and contextual data, and interacting with passengers and nearby road users. The developed platform has been applied in studies addressing passenger monitoring and social interactions inside the shuttle. Its modular architecture enables researchers to investigate different concepts and functions without requiring a fully automated vehicle. The WoZ shuttle therefore provides a practical test environment for developing and evaluating sensor-based assistance systems, optimizing public transport functions, and studying factors that influence the safety, acceptance, and experience of users of future automated public transport.

## 1. Introduction and Related Work

Automated shuttles are a promising approach for shaping the future of public transportation. They are primarily used for first-mile/last-milemobility to improve connectivity between residential areas and public transport [1,2,3,4,5]. This is particularly relevant in rural areas, where the comprehensive use of public transportation still has considerable potential for improvement [3]. Furthermore, automated shuttles can improve mobility and accessibility for individuals who are unable to drive due to demographic or physical factors. The demographic factors include younger people who have not yet obtained a driver’s license, as well as older adults who may no longer hold a driver’s license or have access to a private vehicle [3].

This paper focuses on an experimental platform that operates a shuttle in a real-world environment. PAULA, platform for automated user-centred local mobility analysis, is not a fully automated vehicle but is driven by a human operator, who remains invisible to the passengers due to the physical separation by monitors between the driver’s compartment and the passenger compartment. This Wizard-of-Oz (WoZ) approach [6] enables realistic experiments with passengers in an automated driving environment, allowing investigations of passenger behavior and intentions. The hardware and software of the shuttle are detailed in Section 3 and Section 4. In parallel, an analog twin of the shuttle is available in the laboratory (Section 5), equipped with the same sensors as the real vehicle. It is used to conduct experiments under controlled conditions and for preliminary studies before conducting experiments in the real shuttle. Finally, Section 6 presents some example use cases for the developed WoZ concept.

### 1.1. Acceptance and User Experience of Automated Shuttles

A major challenge in the deployment of automated shuttles is acceptance among passengers and other road users. Acceptance strongly depends on perceived safety, trust in the technology, and interaction between the vehicle and its surroundings [3,4,5,7,8]. In particular, perceived safety has been widely investigated in the literature and is influenced by several factors. Table 1 provides a brief overview of these influencing factors as examined in the literature.

These aspects are relevant for all road users interacting with the automated shuttle. Not only passengers inside the shuttle but also pedestrians, cyclists, and drivers of other vehicles rely on the vehicle’s functionality. The gestures typically used by a human driver to communicate with other road users are absent in automated shuttles. However, they can be replaced by appropriate signals and interaction through human-machine interfaces (HMIs) [5].

### 1.2. WoZ Approaches and Hidden Driver Studies

The WoZ approach enables researchers to investigate passenger interaction with automated shuttles and their behavior without requiring the vehicle to operate autonomously. The manually operated WoZ shuttle also allows users to select from a variety of predefined profiles, ranging from comfortable to sporty to risky. Distler et al. [2] implement an approach by allowing passengers to board an NHTSA Level 4 shuttle, whose supervisor is disguised as another passenger and only takes control in emergency situations. However, the WoZ term is used here for the shuttle booking process, as participants are led to believe they are booking an on-demand automated shuttle. In reality, the operator waits for that moment and manually dispatches the shuttle. A similar approach is adopted by Nordhoff et al. [9], who investigate not only passengers’ perceived safety but also the interaction between the vehicle and crossing-road users as well as the conditions required for effective human-vehicle interaction. In contrast, Meurer et al. [6] investigate passenger behavior and perceived safety in an automated passenger car, where the driver’s seat is completely concealed by a curtain. To initiate the interaction, passengers are instructed to use a predefined wake word. The driver is instructed to respond in the way one would expect from a commercial voice assistant such as Amazon Echo.

### 1.3. Experimental Platforms

Several additional studies provide insights into passenger acceptance and behavior in automated shuttle systems. Avsar et al. [5] employ the SAE Level 4 EasyMile EZ10 shuttle as a first-mile/last-mile shuttle and on-demand service. Its maximum speed is limited to 18 km h^−1^. Besides the passenger experience, the study primarily focuses on general findings regarding usage behavior, the booking process, and service availability. An officially designated onboard operator is present and can take control of the vehicle in emergency situations. Eden et al. [7] and Prencipe et al. [8] also deploy SAE Level 4 shuttles in real-world environments to investigate passenger experience and acceptance. These vehicles likewise operate with an onboard operator along predefined routes at maximum speeds of 20 km h^−1^ and 25 km h^−1^. In addition, Prencipe et al. [8] use the Muse 2 Electroencephalography (EEG) headset (RRID: SCR 014418) [10] to record passengers’ EEG signals. From these signals, they derive metrics and infer indicators associated with attention, stress, relaxation, and specific emotional states. Their results indicate that participants exhibit higher levels of attention and relaxation during the ride. Furthermore, participants remain in a positive emotional state, and no elevated stress levels are detected. However, interviews also reveal that participants prefer the presence of an onboard operator because it increases their perceived sense of safety. Consequently, the findings may differ in scenarios without an onboard operator.

In contrast to these studies, the study by Escher et al. [11] presents a static shuttle mock-up that simulates the passenger compartment of an automated shuttle. The interior design is based on current prototypes like the ones from EasyMile [12] or Navya [13]. The side windows and windshield are replaced with displays and projectors that simulate a real driving experience. Passengers are given the opportunity to contact an operator in specific situations. The study investigates when this option is used and to what extent participants are willing to assist in resolving operational problems. This setup resembles the physical laboratory twin also presented in the paper at hand, which is likewise intended for controlled user studies.

### 1.4. Research Gap: Integrated Human-Centered Experimental Platforms

The reviewed studies demonstrate that different experimental platforms address specific aspects of passenger experience, acceptance, and interaction in automated shuttle systems. Although questionnaires and interviews can capture passengers’ subjective perceptions, such information will not be available during future real-world operation. In addition, these capabilities are generally investigated separately. WoZ approaches enable studies in moving vehicles, while static laboratory mock-ups, such as the platform presented by Escher et al. [11], provide controlled experimental conditions but cannot reproduce the dynamics of a moving shuttle. Other studies incorporate physiological sensing [8] but do not combine it with comprehensive monitoring of passengers and their surroundings, human-machine interaction, and synchronized data acquisition. As a result, no automated response to the individual and situational behavior of passengers or other road users is possible.

The experimental platform presented in this paper addresses this gap by integrating these capabilities within an experimental infrastructure. It combines controlled laboratory experimentation with a moving shuttle and enables synchronized monitoring of passenger states, the surrounding environment, and interactions between the shuttle and its passengers and other road users. Furthermore, the modular setup supports the transfer of investigated scenarios and sensing approaches between laboratory and field studies. In addition, the platform allows the investigation of the effects of higher shuttle speeds in urban and rural areas on passenger experience and acceptance. This combination distinguishes the proposed platform from existing WoZ approaches, automated-shuttle studies, and static laboratory mock-ups. The following Table 2 provides a comparison of the experimental platform presented in this work with existing automated-shuttle research platforms.

## 2. WoZ Shuttle as a Prototyping Platform for Automated Public Transport

The WoZ method is a prototyping approach in which users perceive a system function as automated, although the function is partially or completely performed by a concealed human operator. The method supports the investigation of user behavior, expectations, interaction requirements, and system acceptance before the corresponding technology is fully implemented [14,15]. In automated vehicle research, WoZ configurations enable realistic studies of apparently driverless vehicles while a hidden human driver retains control of the vehicle [16,17]. This approach is particularly suitable for automated public transport, for which a fully automated and freely configurable research shuttle is not available for the intended investigations.

The development of automated public shuttles involves more than automated longitudinal and lateral vehicle control. In the absence of a visible driver or onboard service personnel, the shuttle itself must monitor the passenger compartment, detect potentially safety-critical situations, communicate with passengers, provide appropriate information or assistance and communicate as well as operate together with the control center. It must also perceive the surrounding traffic environment, identify relevant road users, estimate their behavior and intentions, and communicate with pedestrians, cyclists, and drivers of other vehicles. These functions influence perceived safety, trust, usability, and acceptance and therefore require investigation before fully automated public shuttles become widely available [9,18].

To support such investigations, the proposed WoZ shuttle serves as a modular prototyping and experimental platform. The WoZ principle applies primarily to the dynamic driving task: a concealed human driver controls the shuttle, whereas passengers experience the journey without seeing the driver. The driver remains responsible for safe vehicle operation and can respond directly to unexpected traffic situations. In contrast, the passenger monitoring, environmental perception, event detection, intention prediction, and interaction functions operate automatically through the corresponding sensors, algorithms, and interfaces. These functions do not require continuous real-time observation or intervention by a human instructor. This separation between simulated and implemented automation is a central characteristic of the concept. Vehicle guidance represents the simulated automated function, whereas passenger monitoring, environmental perception, and interaction represent functions that are technically implemented and evaluated. The human driver therefore ensures safe and reliable vehicle operation without replacing the algorithms under investigation. This arrangement enables non-driving functions of automated public transport to be developed independently of a complete automated driving system.

**Figure 1:**
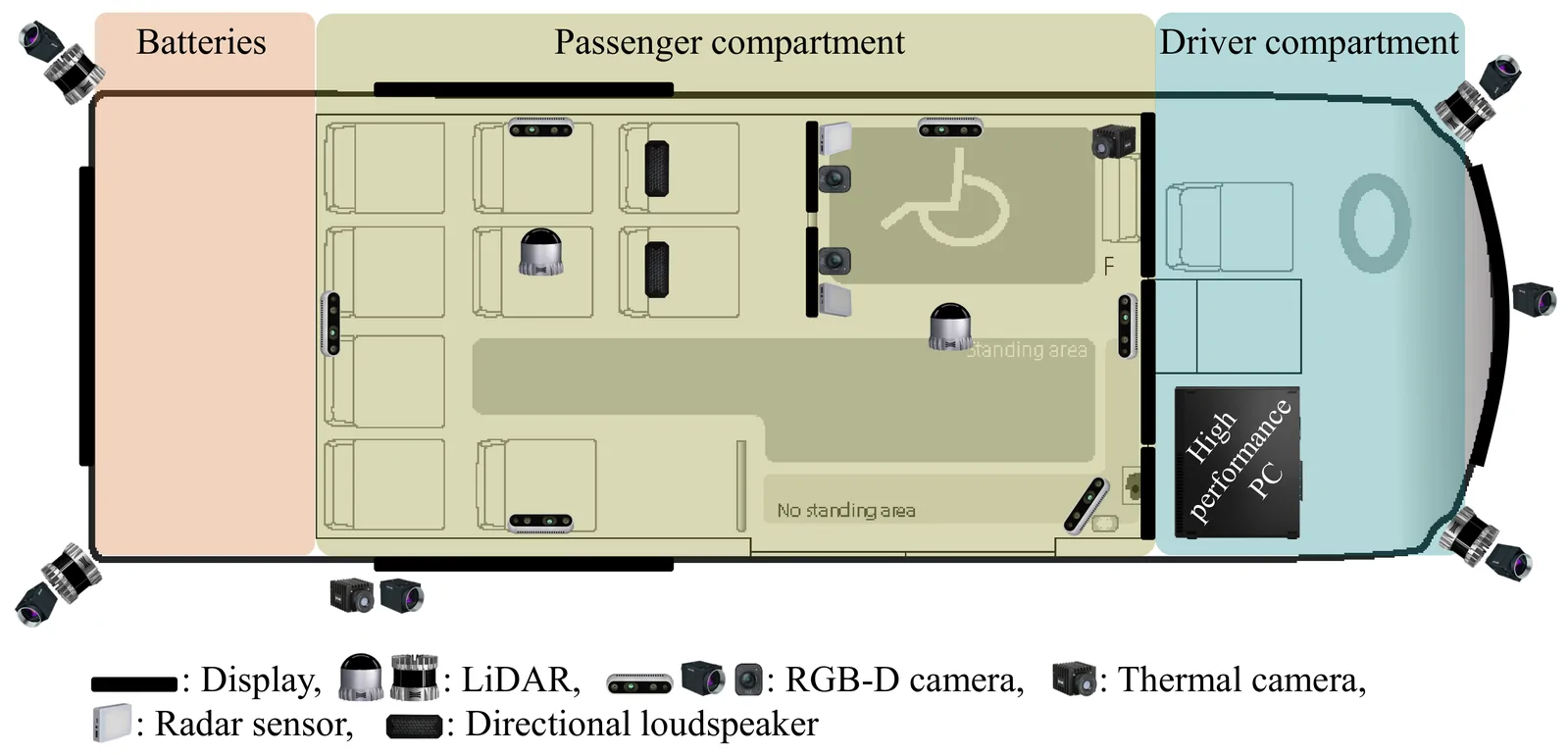
Schematic illustration of the implemented Wizard-of-Oz (WoZ) concept in the shuttle. The driver and passenger compartments are separated by a partition wall incorporating monitors facing the passenger compartment, which display the forward driving scene to the passengers. The shuttle is equipped with interior and exterior monitoring sensors and interaction interfaces to support research on human-shuttle interaction.

The concept is implemented in a shuttle with nine passenger seats. Figure 1 presents the WoZ concept in the shuttle. The shuttle operates as a public transportation service along a predefined route. In accordance with ethical requirements, the presence of a safety driver is not concealed from the passengers; thus, passengers are aware upon boarding that a driver is present on board. However, during the journey, the driver is not visible to the passengers and is not available for direct communication. A wall physically separates the driver compartment from the passenger compartment and prevents passengers from seeing the driver during the journey. A door integrated into the wall provides access to the driver compartment and remains closed during normal experimental operation. Concealing the driver reduces visual and social cues associated with conventional public transport operation. The lack of visual and communicative access to the driver is intended to strengthen the perception of automated driving and to support the development of this impression over the course of the journey. This is methodologically relevant because the visible presence of a driver or safety steward can influence passengers’ perceived safety, trust, attention, and attribution of responsibility [9,17]. The compartment separation does not restrict the driver’s ability to operate the shuttle safely. Instead of conventional side mirrors, the driver uses camera-based views of the lateral areas of the vehicle. A separate camera provides a view of the passenger compartment because the closed separation wall prevents direct observation of the passengers. These camera views allow the driver to maintain awareness of the vehicle surroundings and to respond to situations inside the shuttle that require an operational safety intervention. The driver’s view of the passenger compartment is independent of the automated passenger monitoring functions investigated using the platform.

The separation wall also restricts the passengers’ direct view through the front windshield. Therefore, three monitors are mounted on the wall facing the passenger compartment. During the journey, the monitors present a real-time view of the scene in front of the shuttle. Passengers can consequently observe the road, surrounding vehicles, traffic infrastructure, pedestrians, cyclists, and other relevant events ahead of the vehicle. The displays preserve passenger situational awareness while maintaining the visual separation between the driver and passenger compartments. After a short time on the road, passengers think the vehicle ends at the partition. The view of the traffic area in front of the vehicle provided by the screens also supports the credibility of the automated shuttle experience. This is important because restricted visibility of the traffic situation can produce uncertainty or discomfort that results from the experimental setup rather than from the automated shuttle concept itself [17]. Internal displays can also support the communication of vehicle status, current actions, and intended maneuvers to passengers [19].

The shuttle incorporates multimodal sensing of both the passenger compartment and the external environment. This provides complementary information on visual appearance, depth, spatial geometry, motion, and thermal characteristics. Interior sensing supports the analysis of passengers’ presence, posture, activity, social behavior, mental and emotional state, and potentially safety-relevant events. Exterior sensing supports the detection and tracking of surrounding road users and the prediction of their movements and intentions. Such monitoring capabilities are particularly relevant for automated and shared vehicles because functions traditionally performed by a driver or onboard attendant must be supported by technical systems [20]. The detailed sensor configuration, installation positions, and data acquisition infrastructure are presented in Section 3. In addition to monitoring the interior and exterior environments, the platform supports interaction with passengers and surrounding road users. Visual and auditory interfaces provide information, warnings, instructions, and assistance to passengers inside the shuttle. External interfaces support communication with pedestrians, cyclists, and other traffic participants. Multimodal communication is advantageous because the suitability of a communication channel depends on the type and urgency of the message, environmental conditions, and the recipient’s attention. External communication is especially relevant for automated vehicles because conventional interpersonal cues, such as eye contact and gestures from a driver, are no longer available [21], and it also offers additional features that make the vehicle interconnected with its surroundings. The interface implementation and the associated software functions are described in Section 4.

The platform is designed according to five main requirements:

- Credibility of the automated shuttle experience;
- Operational safety;
- Experimental controllability;
- Suitability for the analysis of scientific datasets;
- Extensibility.

Credibility is supported by concealing the driver and presenting the forward traffic scene to the passengers. Operational safety is maintained through continuous vehicle control by the human driver and camera-based observation of the vehicle interior and surroundings. Experimental controllability results from separating the driving task from the automated functions under investigation. Extensibility is achieved through a modular configuration that supports the integration, replacement, and reconfiguration of sensors, algorithms, and interaction interfaces.

A principal benefit of the platform is the combination of realistic mobility conditions with experimental control. Participants experience an actual journey in a moving shuttle, including acceleration, braking, vibration, environmental noise, traffic interactions, and the presence of other passengers. At the same time, the use of a human driver allows studies to be conducted without depending on the availability or operational limitations of an experimental automated driving system. The platform therefore provides a more realistic environment than a stationary mock-up or desktop simulation while retaining sufficient flexibility for controlled investigations. The platform represents an automated public shuttle experience but does not constitute a fully automated vehicle. The behavior of a human driver can differ from that of an automated driving system with respect to trajectory planning, acceleration, braking, gap acceptance, and reactions to ambiguous traffic situations. Consequently, the platform is not intended to evaluate the performance or safety of an automated driving stack. It instead supports the development and evaluation of passenger-, perception-, monitoring-, assistance-, and interaction-related functions in a simulated driverless context. Participant information, informed consent, privacy, data protection, and any required debriefing are addressed in the protocols of the individual studies.

## 3. Hardware Configuration of the WoZ Shuttle

### 3.1. Vehicle Platform and System Layout

**Figure 2:**
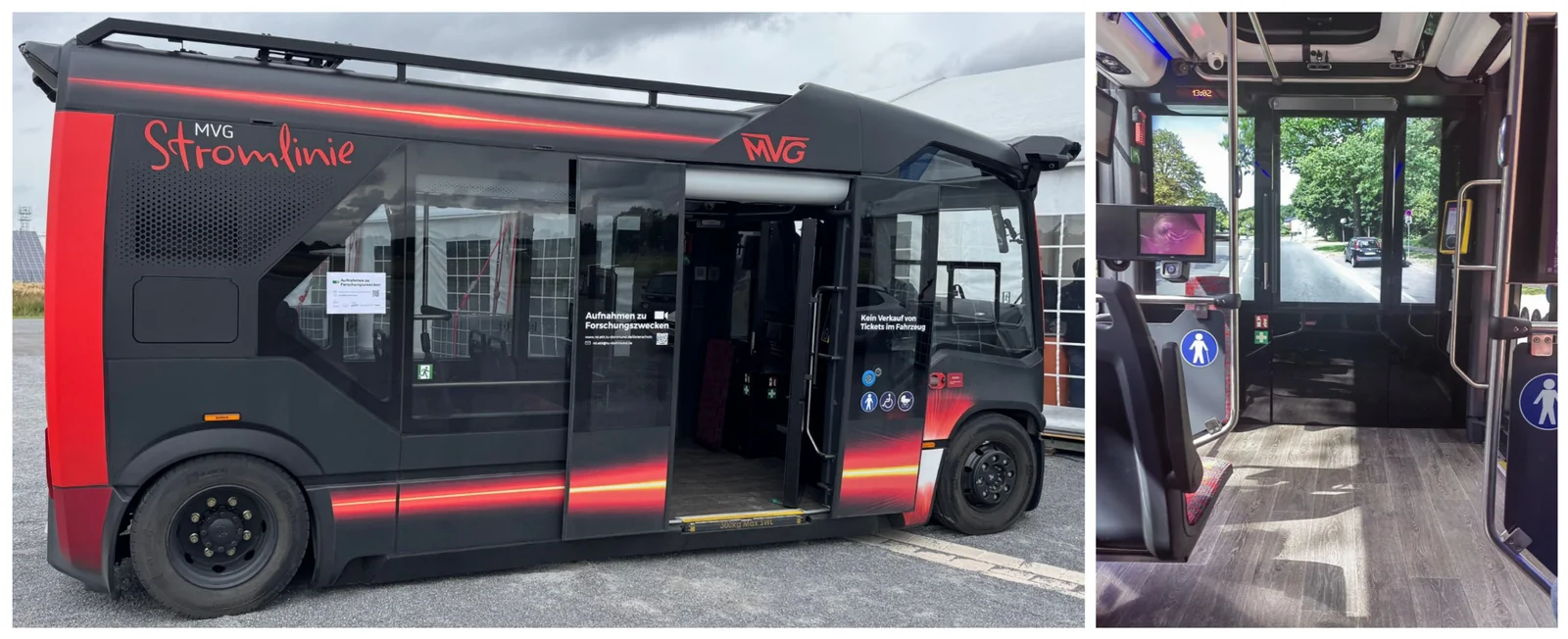
Platform for automated user-centred local mobility analysis (PAULA), the equipped WoZ shuttle regularly used in public transport (**left**), and the passenger compartment view (**right**). Monitors integrated into the partition wall display the forward driving scene to passengers, creating the impression of automated vehicle operation.

The WoZ platform uses an eVersum mobility electro shuttle MK II-400 (eVersum mobility solutions GmbH, Leibnitz, Austria) as its base vehicle [22]. The shuttle has external dimensions of approximately 6.90 m × 2.30 m × 3.10 m in length, width, and height, respectively. The research configuration provides nine passenger seats and contains the physical separation between the driver and passenger compartments described in the preceding section. Figure 2 depicts the passenger view in the WoZ shuttle. The research hardware is organized into four functional groups: whole-compartment interior monitoring and interaction, seat-specific interior monitoring and interaction, external-environment perception and communication, and onboard data processing. In the following description, the term curbside denotes the side of the shuttle containing the passenger entrance, whereas roadside denotes the opposite side facing the traffic lane during normal operation.

### 3.2. Interior Monitoring and Interaction Hardware

Two complementary concepts are available for monitoring and interacting with passengers. The general concept addresses all passengers within the compartment, whereas the individual concept provides seat-specific sensing and interaction for the two adjacent seats in the first passenger row. The general configuration supports investigations involving passenger groups, compartment occupancy, social interaction, mental and emotional state of individuals, and safety-relevant activities. The individual configuration enables detailed investigations of a single passenger’s behavior, physiological state, mental and emotional state, and interaction with the shuttle.

#### 3.2.1. General Interior Monitoring

Whole-compartment visual and depth sensing is provided by two Intel RealSense D456 cameras and four Intel RealSense D435i cameras (Intel RealSense Technology, Cupertino, CA, USA) [23]. One D456 is mounted in the front ceiling area and the other in the rear ceiling area of the passenger compartment. The D456 is a stereo depth camera that provides both depth and RGB data. It offers a depth field of view of approximately 86° × 57°, a specified operating range of approximately 0.6–6 m, and depth-stream configurations with a resolution of up to 1280×720 pixels and a frame rate of up to 90 fps. The camera also incorporates an inertial measurement unit. The front-rear arrangement provides complementary longitudinal views of the passenger compartment.

The four D435i cameras are also mounted in the ceiling area. Two cameras are positioned at the front of the passenger compartment, with one camera on the left and one on the right, and the remaining two are positioned correspondingly at the rear. The D435i uses active stereoscopic depth sensing and incorporates a six-degree-of-freedom inertial measurement unit. Depending on the selected streaming mode, it provides depth data at resolutions of up to 1280×720 pixels and frame rates of up to 90 fps. Its depth field of view is approximately 87° × 58°, while the RGB sensor provides images with a resolution of up to 1920×1080 pixels at 30 fps [23]. The laterally separated front and rear views are intended to reduce occlusions caused by seats, standing passengers, and interactions between passengers.

Thermal imaging of the complete passenger compartment is provided by one FLIR A70 long-wave infrared camera (Teledyne FLIR LLC, Wilsonville, OR, USA) [24] mounted in the front ceiling area. The camera provides a thermal image resolution of 640×480 pixels, operates in the spectral range of 7.5–14 μm, and supports image acquisition at 30 Hz. The thermal stream captures spatial temperature distributions and temporal temperature changes without requiring visible illumination. These data support the analysis of passenger presence and skin temperature patterns that may be associated with physiological or affective responses. The integrated A70 has a field of view of 95°.

Two Ouster OSDome Rev7 LiDAR sensors (Ouster, San Francisco, CA, USA) [25] provide 3D information about passenger motion and use of the interior space. The sensors are spaced along the ceiling and oriented toward the passenger area. The OSDome provides a hemispherical field of view of 180° vertically and 360° horizontally. The Rev7 specification provides a range of up to 20 m for a target with 10% reflectivity under the stated test conditions, a selectable vertical resolution of 128 channels, horizontal resolutions of 512, 1024, or 2048 samples per revolution, and rotation rates of 10 or 20 Hz. The overhead installation uses the hemispherical field of view to capture passenger positions, movements, trajectories, and spatial relationships throughout the compartment.

General interaction with all passengers is provided through the three monitors mounted on the separation wall between the driver and passenger compartments and through a whole-compartment loudspeaker. The monitors present the real-time scene in front of the shuttle and can additionally display vehicle state information, notifications, warnings, or experimental interface content. The loudspeaker provides a common auditory communication channel for announcements and assistance messages intended for all passengers.

#### 3.2.2. Individual Interior Monitoring and Interaction

The first passenger row contains two adjacent instrumented seats for individual-specific studies. Each seat is assigned a radar-based vital-sign-sensing system, a camera, a touchscreen, and a directional loudspeaker. The components are arranged so that the radar and camera observe the occupant, the touchscreen remains accessible to the occupant, and the directional loudspeaker directs auditory information primarily toward the corresponding seat. This configuration enables individualized sensing and interaction while limiting interference with passengers in neighboring seats.

Each instrumented seat incorporates a Sykno ViRa24 continuous wave (CW) radar system (Sykno, Erlangen, Germany) [26] positioned in front of the passenger. The sensor is used to measure the heart rate (HR) and respiratory rate (RR) of the passenger. Detection is based on changes in the phase of the reflected radar signal caused by body movements. The distance relative to an imaginary zero point can be calculated using the formula

(1) $$d=\frac{c\;\cdot \;\varphi }{4\pi f},$$

where c is the speed of light, *f* is the frequency of the radar signal, and φ is the phase shift of the reflected signal. The sensor is positioned approximately 0.3 m from the passenger and has a sampling rate of 1000 Hz. The half-power beamwidth (3 dB beamwidth) is 22° in the horizontal and 17° in the vertical direction relative to the sensor axis. This radar sensor is specifically designed to detect cardiac and respiratory movements. Therefore, the output range is divided into three frequency bands that are optimized for the detection of cardiac and respiratory movements. A two-channel electrocardiography (ECG) system is also integrated into the device as a reference for heart activity measurements. To determine the HR, RR, and heart sound the following frequency bands are used [27,28,29].

- RR: 0.1 to 0.5 Hz (6 to 30 bpm);
- HR: 0.8 to 2.5 Hz (48 to 150 bpm);
- Heart sound: 15 to 60 Hz.

Three ECG electrodes must be attached to the subject being measured to record the typical waveform of the cardiac cycle. Two of them are attached to the left and right sides of the heart, below the collarbone. The third electrode is placed on the lower back [26]. For optimal measurements with the radar sensor, it is important that the sensor axis be oriented perpendicular to the chest of the subject being measured and positioned approximately at heart level to detect cardiac and respiratory movements as effectively as possible. In addition, the person should remain as still as possible to avoid motion artifacts.

A respiration belt, Go Direct Respiration Belt (Vernier, Beaverton, ON, USA) [30], is used as a contact sensor for ground-truth measurement of the RR. It is worn around the chest and measures the strain caused by respiration, which is transferred through the belt to a force sensor. The resolution of this sensor is 0.01 N, and the data are recorded at a sampling rate of 10 Hz. A similar configuration is also used in previous studies [31]. The extraction of the RR can be performed using application-specific signal processing algorithms.

A Logitech StreamCam [32] provides seat-specific RGB video. The camera records Full HD video with a resolution of 1920×1080 pixels at up to 60 fps, provides a 78° field of view, and includes automatic focusing and exposure functions. The camera is oriented toward the passenger to capture facial appearance, head orientation, upper-body motion, gestures, and interactions with the touchscreen.

Visual interaction at each seat is provided by a touchscreen monitor [33]. The monitor has a 0.25 m active matrix thin-film transistor liquid crystal display, a native resolution of 1280×800 pixels, a 16:10 aspect ratio, and projected capacitive sensing for up to ten simultaneous touch points. The touchscreens can support individualized questionnaires, information presentation, assistance requests, entertainment functions, and interactive HMI studies.

Individual auditory interaction is implemented using Molitor Audfly FS mini directional ultrasonic loudspeakers [34]. Each unit contains an integrated amplifier and provides a specified audio frequency range of 500 Hz to 12 kHz. Its directional pattern is narrower than 15°, measured at the −20 dB level, and its specified maximum sound pressure level is 74 dB at 1 kHz and a distance of 2 m. The enclosure measures 170×70×45 mm^3^ and operates from a 12 V, 2 A supply. By directing the loudspeaker toward its associated seat, different auditory messages can be presented to individual passengers with reduced acoustic exposure of the remaining passenger compartment.

### 3.3. External-Environment Monitoring

The external sensing system combines LiDAR, visible spectrum cameras, and thermal imaging. The sensors are distributed along the upper edges and corners of the shuttle to provide broad coverage of the surrounding traffic environment while minimizing obstruction by the vehicle body.

Two Ouster OS1 sensors (Ouster, California, U.S.) [35] are mounted along the upper edge on the roadside of the shuttle. The OS1 is a mid-range rotating LiDAR with a 360° horizontal field of view. The Rev7 specification provides a vertical field of view of approximately 42.4°, a range of up to 90 m for a target with 10% reflectivity under the specified test conditions, a resolution of 128 vertical channels, and selectable rotation rates of 10 or 20 Hz. The roadside placement supports the detection and tracking of vehicles and other road users in adjacent traffic lanes and at larger distances.

Two Ouster OS0 sensors (Ouster, California, U.S.) [36] are mounted correspondingly along the upper edge on the curbside. The Rev7 OS0 provides a 360° horizontal field of view and an approximately 90° vertical field of view. Its specified range for a target with 10% reflectivity is up to 35 m, and it supports 128 vertical channels and rotation rates of 10 or 20 Hz. The wider vertical field of view is suitable for near-field monitoring of pedestrians, cyclists, the boarding area, and other objects located close to the side of the shuttle. The combined OS1 and OS0 configuration therefore provides complementary longer-range roadside coverage and wide-angle curbside coverage.

A second FLIR A70 thermal camera is mounted on the upper rear part of the curbside vehicle body and is directed toward the passenger entrance. It provides thermal observations of persons approaching, entering, or leaving the shuttle. Its 640 × 480 pixel long-wave infrared (LWIR) stream complements the visible spectrum and 3D measurements in the entrance area, particularly under low light conditions.

Visible spectrum external monitoring is provided by six Teledyne FLIR Blackfly S GigE cameras (Teledyne Vision Solutions, Milpitas, CA, USA) [37]. Four cameras are mounted at the four upper corners of the shuttle. An additional camera is installed on the upper rear part of the curbside to monitor the entrance area. All five cameras are equipped with a ruggedized 6 mm, 5 megapixel lens designed for sensors with a format of up to 0.03 m and for environments subject to shock and vibration [38]. Its short focal length provides wide-angle coverage and is therefore used for the four corner views and the entrance-monitoring view. The sixth camera is mounted on the upper front surface of the shuttle and uses a compact 12 mm, 5 megapixel lens [39]. Its longer focal length provides a narrower forward-facing view than the 6 mm lenses and supports the observation of road users and traffic infrastructure ahead of the shuttle.

The Blackfly S GigE camera family supports GigE Vision connectivity and IEEE 1588 [40] Precision Time Protocol functionality, which facilitates integration into Ethernet-based multi-camera systems. The sensor has a resolution of 2448 × 2048, a pixel size of 3.45 μm, and a maximum frame rate of 24 fps.

### 3.4. External Visual Interface

Visual communication with pedestrians, cyclists, and drivers of surrounding vehicles is provided by RGB LED matrix assemblies installed on the front, rear, curbside, and roadside surfaces of the shuttle. Each side-mounted assembly measures 192×960 mm^2^ and consists of five horizontally arranged 64×64 RGB LED matrix modules. Each module measures 192×192 mm^2^, contains 4096 RGB pixels, and has a pixel pitch of 3 mm. The resulting assembly provides a resolution of 64×320 pixels on each side of the vehicle.

The individual matrix modules have a manufacturer-specified brightness of 2800 cd m^−2^ and a refresh frequency of at least 400 Hz [41]. The four-sided arrangement enables the shuttle to present status information, warnings, symbols, or intended maneuver information to road users approaching from different directions. The displayed content and control of the matrix assemblies are described as part of the software and interaction architecture.

### 3.5. Onboard Computing Hardware

All sensor streams and interaction devices are connected to two onboard workstations. Both computers are installed in the driver compartment on the right-hand side of the driver, where they remain physically separated from passengers while remaining accessible for system supervision and maintenance. The sensing architecture includes both USB-based devices, such as the RealSense cameras, StreamCams, touchscreens, and vital sign radars, and Ethernet-based devices, including the Ouster LiDAR sensors, FLIR thermal cameras, and Blackfly S GigE cameras.

Each workstation incorporates NVIDIA RTX 6000 GPUs (single and double) and a Samsung 990 PRO 4 TB M.2 NVMe solid-state drive. The SSD uses a PCIe 4.0×4 interface and the NVMe 2.0 protocol. The manufacturer specifies sequential read and write rates of up to 7450 and 6900 MB s^−1^, respectively [42]. The two GPU-equipped workstations provide the processing and storage capacity required for concurrent multi-sensor acquisition, online perception, passenger monitoring algorithms, interaction control, and high-volume experimental data recording. The allocation of individual sensors and algorithms to the two workstations, as well as the data synchronization and communication architecture, is presented in the software section.

## 4. Software and Algorithms

### 4.1. Implementation Framework for Hardware Integration and Data Acquisition

The shuttle is equipped with several different sensors for passenger monitoring and displays for enabling human-machine interaction (see Section 3). The goal is to integrate all hardware into a robust and locally secured framework, which is independent of system changes or operating system (OS) updates. For that purpose, the experimental platform is containerized using Docker [43]. Containerization is particularly suitable since the software stack typically depends on a heterogeneous combination of OS packages, ROS 2 middleware, sensor drivers, perception libraries, GPU-accelerated frameworks, and the project-specific source code. Instead of installing these dependencies directly on each host computer, Docker packages the required runtime environment into an image that can be instantiated as a container isolated from the host’s OS. In contrast to a full virtual machine, a container shares the kernel of the host OS and therefore introduces comparatively low overhead while still isolating the user-space environment. This reduces dependency conflicts, simplifies deployment on multiple machines, and improves the traceability of the experimental setup by making the software environment explicitly versioned and repeatable [44]. For the present system, this is advantageous because sensor processing, visualization, and data recording can be executed close to native performance while preserving a reproducible development and deployment workflow.

For interactive development and experiment execution, the Docker workflow is extended using rocker, a Docker wrapper from the Open Source Robotics Foundation [45] that simplifies the configuration of host-dependent container features, such as X11/GUI forwarding, user-ID mapping, mounted workspaces, and NVIDIA GPU access. These features are important for the presented setup because several components require graphical tools, while other components require hardware-accelerated processing for perception and neural-network inference. Therefore, rocker reduces configuration complexity and improves the robustness of the development workflow, especially when the same container image is used across multiple host computers.

The higher-level automated driving software structure follows the modular design principles of Autoware [46], which is an open-source software stack that provides functionality for the main processing stages of an automated vehicle, including sensing, localization, perception, planning, control, system monitoring, and data collection [47]. Its architecture is based on Robot Operating System 2 (ROS 2) interfaces and separates the driving task into replaceable modules rather than implementing the complete autonomy pipeline as a monolithic program [46]. This modularity is beneficial for experimental platforms (e.g., [48]) because individual components can be enabled, disabled, exchanged, or extended depending on the experimental objective and is therefore well suited for the presented multi-sensor evaluation platform.

The distributed software architecture of the experimental platform is implemented using ROS 2 Humble Hawksbill [49]. ROS 2 provides a modular robotics middleware in which individual software components are implemented as nodes that communicate through topics, services, and actions. This publish-subscribe structure is well suited for multi-sensor experimental platforms because hardware drivers, synchronization modules, perception algorithms, visualization tools, and data recorders can be developed and executed as separate processes while still exchanging standardized messages through a common communication layer [50]. The use of ROS 2 Humble is additionally motivated by its long-term-support character and its official support for Ubuntu 22.04, which makes it suitable for the presented research setups, which must remain stable over the duration of data collection and evaluation [49].

Timestamp handling is a central requirement for synchronized multi-sensor data collection. ROS 2 provides a time abstraction that distinguishes between system time, steady time, and ROS time, thereby supporting both real-time hardware operation and replay or simulation workflows [49]. For hardware drivers, timestamps are assigned close to the acquisition time and transported with the corresponding message headers. Downstream modules then associate data from different sensors using these timestamps instead of requiring all sensors to operate at an identical sampling rate. In the presented setup, synchronization therefore refers to timestamp-based temporal association rather than hardware-triggered frame synchronization. The raw sensor streams are recorded with their original timestamps and sensor-specific acquisition rates, and no additional resampling or forced frame alignment is applied during data collection. The achievable synchronization accuracy is consequently determined by the timestamp accuracy of the individual drivers, the clock synchronization between the host systems, and the temporal tolerance used for later message association. For post-processing or online fusion, temporally corresponding measurements can be selected by minimizing the timestamp difference or by using an approximate-time synchronization window. The remaining synchronization error can be quantified as the timestamp spread between the associated messages. This approach is appropriate for the presented human-centred measurement setup, since the relevant behavioural dynamics evolve on the scale of several hundred milliseconds to seconds, while the timestamp-based association preserves the original timing information of each sensor stream.

Experimental data is recorded using an adaptation of rosbag2 and the MCAP storage format. A recorded bag consists of timestamped messages that can later be replayed for debugging, evaluation, visualization, and algorithm development [49]. The MCAP storage plugin extends rosbag2 with support for the MCAP file format [51]. MCAP is an open container format for multimodal log data that supports multiple timestamped channels, pre-serialized message data, optional compression, indexing, and self-contained schema information [51]. These properties are advantageous for large-scale sensor recordings because they reduce the overhead of handling many independent files, support efficient sequential writing during experiments, and simplify later inspection, conversion, and playback. To avoid data loss and storage overflow, the recording nodes are configured to split the files during runtime into multiple MCAP files with a fixed size of 5 GB each, which allows the experiment to run continuously for several hours without requiring manual intervention. In the case of a full hard drive, the recording nodes switch to another storage device, which is mounted as a separate volume in the containerized environment. In this setup, the system allows continuous recording of multi-sensor data streams for several hours without requiring manual intervention, using up to 14 TB of storage, which is the maximum capacity of the current experimental platform. To reduce storage overhead, the recorded data is compressed using the compression pipeline provided by the Autoware software stack [46], which applies lossless compression to the recorded sensor streams while retaining the original timestamps and metadata. The topic traffic is measured at the application level by a ROS 2 monitor node that subscribes to the configured topics in raw mode and accumulates the serialized payload size for each received message. The reported value of approximately 250MiB/s therefore represents the aggregated ROS 2 payload throughput observed by the subscriber, not the complete physical Ethernet traffic. This payload rate corresponds to a theoretical continuous recording duration of approximately 14.8h. In practice, the usable duration is considered to be slightly lower due to file-system overhead, MCAP metadata, indexing, and storage-reserve margins.

Python 3.10 [52] is used for a substantial part of the hardware integration and experiment-control software through the ROS 2 Python client library rclpy, which provides the canonical Python interface to ROS 2 and allows nodes, publishers, subscribers, timers, parameters, and service interfaces to be implemented using idiomatic Python constructs [50]. This is particularly beneficial for experimental platforms because Python enables rapid prototyping, concise integration of vendor software development kits (SDKs), and direct interaction with the scientific computing ecosystem. At the same time, computationally intensive components can remain implemented in C++, CUDA, or optimized native libraries and still be integrated into the same ROS 2 graph.

The combination of ROS 2 Humble, Python-based integration nodes, and MCAP-based recording therefore provides a practical compromise between flexibility, reproducibility, and performance. Such a combination is also employed in other experimental systems, such as the one developed by Albers et al. [48,53].

### 4.2. Hardware Integration

#### 4.2.1. Intel RealSense Cameras

The RGB-D cameras are integrated into the ROS 2 environment using the Intel RealSense SDK 2.0. The SDK provides the low-level interface for accessing the camera streams and supports integration with ROS and ROS 2, which allows color, depth, infrared, camera-information, and optional inertial measurements to be published as standardized ROS 2 messages [54]. This enables the RGB-D data to be directly synchronized, recorded, visualized, and processed together with the remaining sensor modalities in the common ROS 2 computation graph.

#### 4.2.2. FLIR A70 Long-Wave Infrared Cameras

The FLIR A70 LWIR cameras are connected using the Spinnaker SDK and its corresponding ROS 2 camera driver. Spinnaker provides the vendor interface for Teledyne FLIR GigE Vision and USB3 Vision cameras, while the ROS 2 driver exposes the acquired image stream as ROS 2 image messages [55]. Since the thermal camera provides radiometric image data, the raw camera output is converted into temperature values using the temperature-measurement formulation provided by Teledyne FLIR, which accounts for the radiometric camera signal and relevant thermal measurement parameters such as object radiation, reflected radiation, atmospheric effects, and calibration constants [56]. The resulting thermal stream can therefore be processed in the same timestamped ROS 2 pipeline as the RGB-D and LiDAR data. The raw radiometric image values *S* are converted into temperature values using the radiometric conversion model provided by Teledyne FLIR [56]. First, the raw pixel value is converted into a camera radiance value by applying the global offset $J_{0}$ and global gain $J_{1}$:

(2) $$L_{\mathrm{data}}=\frac{S-J_{0}}{J_{1}}.$$

The atmospheric water-vapour term $H_{2}O$ is calculated from the relative humidity *h* and the atmospheric temperature $T_{\mathrm{atm}}$ in degrees Celsius:

(3) $$H_{2}O=h\;\cdot \;\exp\left(1.5587+0.06939T_{\mathrm{atm}}-2.7816\times 10^{-4}T_{\mathrm{atm}}^{2}+6.8455\times 10^{-7}T_{\mathrm{atm}}^{3}\right).$$

The atmospheric transmission τ is then computed based on the object distance *d* as

(4) $$\tau =X\exp\left[-\sqrt{d}\left(\alpha_{1}+\beta_{1}\sqrt{H_{2}O}\right)\right]+\left(1-X\right)\exp\left[-\sqrt{d}\left(\alpha_{2}+\beta_{2}\sqrt{H_{2}O}\right)\right].$$

The correction term $K_{2}$ accounts for reflected radiation, atmospheric radiation, and external-optics radiation:

(5) $$K_{2}=r_{1}+r_{2}+r_{3},$$

with

(6) $$r_{1}=\frac{1-\varepsilon }{\varepsilon }\frac{R}{\exp\left(\frac{B}{T_{\mathrm{refl}}+273.15}\right)-F},$$

(7) $$r_{2}=\frac{1-\tau }{\varepsilon \tau }\frac{R}{\exp\left(\frac{B}{T_{\mathrm{atm}}+273.15}\right)-F},$$

and

(8) $$r_{3}=\frac{1-\tau_{\mathrm{ext}}}{\varepsilon \tau \tau_{\mathrm{ext}}}\frac{R}{\exp\left(\frac{B}{T_{\mathrm{ext}}+273.15}\right)-F}.$$

Finally, the object temperature $T_{\mathrm{obj}}$ in degrees Celsius is obtained as

(9) $$T_{\mathrm{obj}}=\frac{B}{\ln\left[\frac{R}{\left(\frac{L_{\mathrm{data}}}{\varepsilon \tau }-K_{2}\right)}+F\right]}-273.15.$$

For the implemented setup, the camera-specific calibration parameters are provided by the manufacturer, while the global object and environmental parameters are set to

(10) $$\begin{array}{cccccccc} \varepsilon & =0.95, & h & =0.5, & d & =1\;m, & & \\ T_{\mathrm{atm}} & =\;{20\;}^{\circ }C, & T_{\mathrm{refl}} & =\;{20\;}^{\circ }C, & T_{\mathrm{ext}} & =\;{33.9\;}^{\circ }C, & \tau_{\mathrm{ext}} & =1. \end{array}$$

The global parameters are chosen to reflect the interior environment of the shuttle, where the relative humidity is around 50%, and the ambient temperature is around 20 °C. The external temperature is set to 33.9 °C to correspond to the human body temperature of 37 °C, which is the relevant temperature for passenger monitoring. The emissivity is set to 0.95, while the object distance is set to 1 m, which corresponds to the approximate minimal distance between the camera and the passengers in the shuttle interior. These parameters are not optimized for absolute temperature measurement, but are rather chosen for relative temperature differences, which is sufficient for the intended passenger monitoring application.

#### 4.2.3. FLIR Blackfly S GigE Cameras

Similarly to the FLIR A70 cameras, the FLIR Blackfly S GigE cameras are integrated using the Spinnaker SDK and its corresponding ROS 2 camera driver [55].

#### 4.2.4. Ouster 3D LiDAR

The Ouster 3D LiDAR sensors (2× OSDome, 2× OS0, 2× OS1) are integrated using the official Ouster ROS driver. After connecting to the sensor, the driver decodes the incoming LiDAR and IMU packets and publishes the corresponding messages, including the 3D point cloud and inertial data, as well as depth images and metadata [57]. As explained in Section 3, the OSDome LiDAR sensors are mounted inside the shuttle, while the OS0 and OS1 sensors are mounted on the shuttle’s body, facing outwards. The OSDome sensors are used for interior passenger monitoring, while the OS0 and OS1 sensors are used for exterior environment perception, exclusively. For the presented sensing setup, the raw point clouds are subsequently processed with the Autoware point cloud preprocessing stack. In particular, the crop-box filter [46] is used to restrict the point clouds by the spatial dimensions of the shuttle and to remove points outside the relevant measurement volume. This reduces the data before downstream perception, tracking, and visualization, increasing the overall system performance and responsiveness, as well as reducing the storage requirements for the recorded data.

#### 4.2.5. CAN Bus Evaluation

The shuttle’s Controller Area Network (CAN) bus is connected to the ROS 2 environment using the ros2_socketcan driver, which provides a bridge between the Linux SocketCAN interface and ROS 2 topics [58]. The driver subscribes to the CAN bus and publishes the received messages as standardized ROS 2 messages, while also allowing outgoing messages to be sent from ROS 2 nodes to the CAN bus. This enables the shuttle’s internal state, such as vehicle dynamics, driver behavior, and other vehicle signals, to be monitored and recorded in synchronization with the multi-sensor perception data.

#### 4.2.6. Sykno ViRa24 Radar Sensor

For the processing of the radar signals acquired by the Sykno ViRa24, the Python-based software provided by the manufacturer is used. The system provides high-resolution in-phase (I) and quadrature (Q) components of the 24 GHz CW radar signal with a sampling rate of 1 kHz. Based on these two signal components, the phase information of the received radar signal is determined while accounting for the respective DC offsets. Phase unwrapping is subsequently applied to compensate for ambiguities caused by phase changes exceeding multiples of 2π. The resulting phase information is then converted into relative changes in the distance between the radar sensor and the subject’s body surface. These distance variations represent mechanical movements of the chest caused by respiration, cardiac activity, and, depending on the measurement configuration, cardiac valve movements [26].

The subsequent signal processing is based on digital filtering of the derived distance signal s to extract the frequency components associated with the respective physiological processes. The software provided by the manufacturer includes a graphical user interface for displaying and storing the acquired raw data, as well as the I/Q signals, target motion, respiratory motion, cardiac motion, and, where applicable, heart sounds. Furthermore, the filter parameters and system settings can be configured by the user. The software also supports the visualization and storage of externally acquired reference signals, such as ECG signals, enabling synchronized comparison with the radar-derived signals. In addition, an automatic compensation of the DC component can be applied during signal processing [26].

It should be noted that the manufacturer-provided software primarily supports the processing of the radar signal up to the extraction of the relevant motion components. The subsequent determination of the HR and RR can be performed using application-specific signal processing algorithms.

### 4.3. Sensor Calibration

#### 4.3.1. RGB Camera Calibration and Denoising

The intrinsic calibration of the RGB cameras is carried out once before data collection using a planar checkerboard calibration target and OpenCV’s calib3d module [59]. The checkerboard contains 7×10 inner corners with a square size of 25mm, resulting in known three-dimensional object points on a planar calibration rig. For each camera, multiple images of the checkerboard are acquired from different poses, the corner locations are detected in image coordinates, and the camera matrix as well as the lens-distortion parameters are estimated using the standard planar camera calibration approach introduced in [60]. Since the cameras are rigidly mounted and the study focuses on human-centred behavioral analysis rather than high-precision driving tasks, the calibration is not repeated before every measurement run. Small variations in the estimated intrinsic parameters are considered negligible with respect to the intended analysis of passenger behaviour, body orientation, and interaction patterns. To improve image quality in challenging illumination conditions, a simple offline denoising step is additionally evaluated using RIDNet, a real-image denoising network based on feature attention [61]. In our measurements, RIDNet improves the signal-to-noise ratio by approximately 3dB under low illumination and by approximately 1dB under high illumination.

#### 4.3.2. LiDAR Calibration

The extrinsic calibration of the LiDAR sensors is performed using an inertial measurement unit (IMU)-assisted point-cloud registration pipeline. The approach is based on the observation that direct registration of raw point clouds can be unstable when the sensors have substantially different mounting orientations or limited initial overlap. Therefore, synchronized point-cloud pairs and representative IMU measurements are first extracted from the ROS 2 data streams. The IMU-derived gravity vectors are transformed into the corresponding LiDAR frames using the available TF tree and used to temporarily level the point clouds to a common vertical direction. This inertial pre-alignment provides a physically plausible initialization but does not replace the geometric calibration step. After preprocessing, including invalid-point removal, range filtering, voxel downsampling, outlier removal, and normal estimation, the relative LiDAR pose is refined using the generalized iterative closest point algorithm introduced in [62]. This procedure follows the general idea of using IMU-based orientation information to condition multi-LiDAR calibration before geometric refinement [63].

#### 4.3.3. Depth, LWIR and Radar Calibration

No additional intrinsic calibration is carried out for the depth modules, the LWIR cameras, or the vital-parameter radar sensors. Instead, the factory-provided calibration parameters of the respective devices are used. This approach is sufficient for the present human-centred platform, where the sensor data are primarily used to capture behavioral patterns, spatial relations, and physiological trends rather than to perform high-precision geometric or physiological reconstruction.

### 4.4. Development of a Multi-Sensor Perception and Interaction Pipeline

#### 4.4.1. Interior Human-Machine Interaction

The shuttle’s interior is equipped with two Elo touchscreens and two directional speakers, enabling rich, individual interactions. The audio-visual HMI is implemented as a Python-based Kivy application and embedded into the ROS 2 software environment. Kivy is an open-source software library enabling rapid development and is selected because it provides a flexible framework for interactive graphical user interfaces in Python [64]. Within the presented system, the HMI is embedded into the ROS 2 framework and subscribes to perception and tracking topics. The visual display and auditory feedback can be updated according to the current scene interpretation, while also considering the user’s input via the touchscreens. This design decouples the user interface logic from the perception pipeline, where object detection, tracking, and situation assessment are performed by dedicated ROS 2 modules, while the HMI only consumes their output and translates relevant events or states into user-facing visual symbols, messages, or sounds. This HMI design further enables the integration of automatic questionnaires, surveys, or other user-interaction modules into the shuttle’s interior environment, which can be used for passenger feedback, user studies, or other human-centered evaluations.

#### 4.4.2. Exterior Human-Machine Interaction

The shuttle is equipped with a set of exterior displays, which can be used to communicate the shuttle’s current state, upcoming maneuvers, and departure times, as well as enable interaction with pedestrians, cyclists or other road users. Each display is connected to a dedicated Raspberry Pi 5|4 GB, which is connected to the central onboard computer via a local area network. The external HMIs are implemented as Python-based server-client applications, where a local message server is instantiated on each Raspberry Pi, while the central onboard computer acts as a client that sends relevant messages to the external displays. The client is embedded into the ROS 2 environment and subscribes to perception and tracking topics, which allows the displays to be updated according to the current scene interpretation.

#### 4.4.3. Perception Pipeline

**Figure 3:**
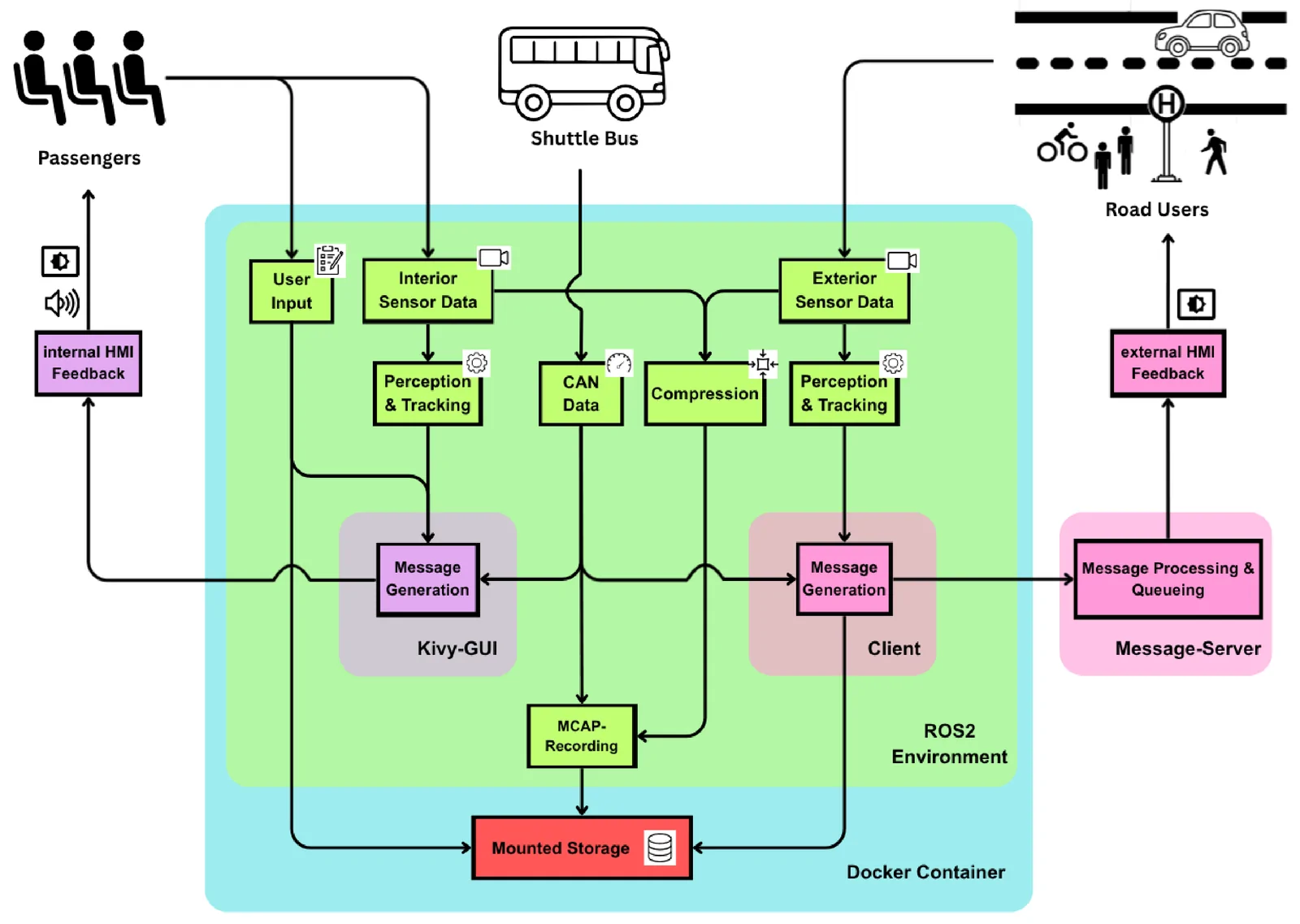
Overview of the implemented software architecture. The multi-sensor perception and interaction pipeline is implemented as a modular ROS 2 graph, embedded into a containerized environment, where each sensor stream can be processed by dedicated nodes. Embedded into the ROS 2 environment, the HMI subscribes to perception and tracking topics and translates relevant events or states into user-facing visual symbols, messages, or sounds. Sensor data and derived perception outputs are recorded using the MCAP storage format, which allows for synchronized replay and analysis of the multi-sensor data streams.

The perception pipeline is implemented as a modular ROS 2 graph, where each sensor stream can be processed by dedicated nodes that perform filtering, object detection, tracking, and situation assessment. The RGB-D cameras provide color and depth images, which can be processed using deep-learning-based object detection algorithms to identify passengers and other relevant objects in the shuttle interior (e.g., [65,66]). Detected objects can be tracked within the scene using dedicated tracking algorithms (e.g., [67]). The LiDAR point cloud can be processed to extract 3D spatial information, yielding rich geometric information of the scene, like occupancy, poses, orientations and Euclidean distances. The thermal camera provides temperature measurements, which can be used to assess passenger physiological responses [68] or detect affective states [69]. A comprehensive overview of the implemented software architecture is provided in Figure 3, which illustrates the modular design, sensor integration, and data flow within the ROS 2 environment.

### 4.5. Latency and Performance Evaluation of the Multi-Sensor Perception Pipeline

Latency is evaluated at the application level by comparing the subscriber-side ROS time with the timestamp contained in each received message. The test is conducted in the emulator and has lasted approximately 30 s. Since the shuttle and the emulator share an identical system architecture, the test is not repeated in the shuttle. For standard sensor messages, the message age is computed as $t_{\mathrm{callback}}-t_{\mathrm{header}}$. For the LiDAR streams, the original point-cloud header timestamps are not used because they are generated in the sensor time domain. The Autoware PublishedTime debug topic is used, and the publication age is computed as $t_{\mathrm{callback}}-t_{\mathrm{published}}$. Additionally, the ROS 2 message rates are evaluated. The message rate describes how frequently messages are received, whereas the message age describes how old a message is when it reaches the subscriber. Thus, a stream can maintain a high rate while still exhibiting high latency if the messages are delayed before publication or during transport. Consequently, both metrics are evaluated. In addition, a ping-pong test is used to estimate the general ROS 2 communication overhead by measuring the round-trip time between a monitor node and a responder node. Table 3 summarizes the grouped sensor latencies before and during MCAP recording, while Table 4 summarizes the corresponding ping-pong baseline. Since the radars are intended to be used in specified experiments, they are excluded from this evaluation. Overall, MCAP recording increases the timing variability of the system, which is most visible in the 95th percentiles. The strongest impact is observed for the compressed LWIR streams and the RealSense depth streams, where the latter also show a clear throughput reduction from approximately 20.4Hz to 11.2Hz. In contrast, the Blackfly cameras and LiDAR publication topics remain comparatively low-latency, although both also show increased tail latencies during recording. The results of the ping-pong test indicate that MCAP recording affects not only sensor-specific processing but also the general ROS 2 communication and scheduling environment. Although some streams operate at comparatively low rates, they remain suitable for the intended human-centred research because the investigated behaviours, such as waiting, boarding, alighting, body orientation, and interaction patterns, evolve on the scale of several hundred milliseconds to seconds rather than within a few milliseconds. Therefore, the achieved sensor rates and latencies are sufficient to capture the relevant behavioural dynamics, while higher rates would mainly improve fine-grained motion reconstruction rather than the interpretation of passenger intentions and interactions.

The cross-modal temporal alignment of the streams is estimated from a representative subset of RGB, depth, and LiDAR sensor timestamps recorded over approximately four minutes during MCAP data recording. For each reference timestamp, the temporally nearest messages from the remaining sensor streams are selected, and the residual inter-sensor timestamp spread is computed as

(11) $$\Delta t_{\mathrm{sync}}=\max_{i}\left(t_{i}\right)-\min_{i}\left(t_{i}\right),$$

where $t_{i}$ denotes the timestamp of the *i*-th sensor message within one associated cross-modal sample. Based on this representative subset, the resulting RGB-depth-LiDAR timestamp spread is 36.53 ms at the median, 46.55 ms at the 95th percentile, 48.56 ms at the 99th percentile, and 59.52 ms at the maximum. These values describe the timestamp-based temporal association accuracy of the cross-modal system and not hardware-triggered synchronization accuracy. In addition, the inter-host time spread can be assessed separately by logging the clock offsets of the acquisition computers, for example, using chrony [70]. However, this inter-host measurement is not yet included in the present analysis.

## 5. Laboratory Emulator as a Purpose-Specific Analog Twin of the WoZ Shuttle

**Figure 4:**
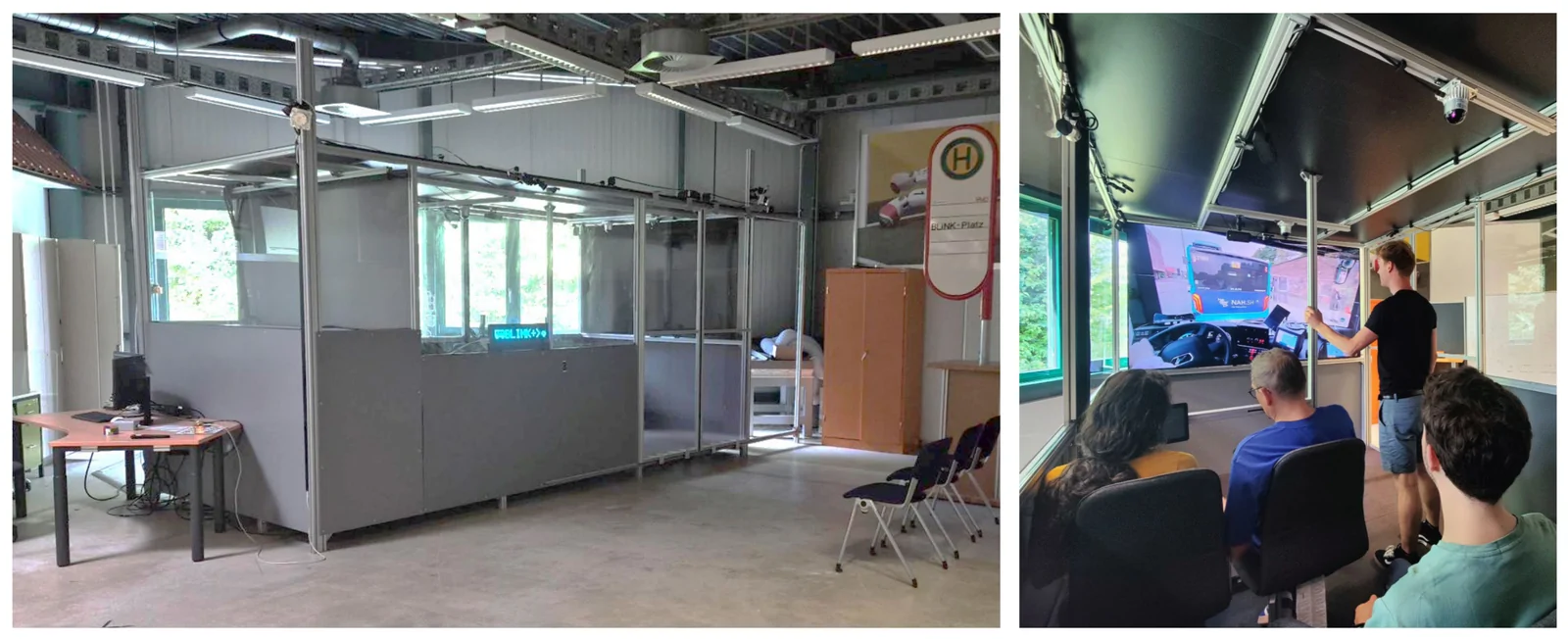
Laboratory emulator of the shuttle passenger compartment. A large front display presents the driving scene during emulated rides. The emulator is equipped with sensors and interaction interfaces corresponding to those installed in the shuttle and supports preliminary experiments as well as studies of safety critical situations. An operator positioned behind the emulator supervises the experiments.

The laboratory emulator (see Figure 4) serves as a purpose-specific physical analog of the WoZ shuttle and supports controlled subject studies of interactions between an automated shuttle and its users. The investigated users include passengers inside the shuttle, pedestrians in the vicinity of the vehicle, and prospective passengers who approach the shuttle, wait at a bus stop, or intend to board. The emulator reproduces the passenger-related experimental environment of the WoZ shuttle but does not constitute a complete replica of the vehicle.

The emulator contains a full-scale reconstruction of the passenger compartment. Its internal dimensions correspond to those of the WoZ shuttle, and nine passenger seats are installed at the same relative coordinates as in the real vehicle. This correspondence preserves the distances between the passengers, sensors, interaction interfaces, and surrounding compartment structures. The supporting structure is constructed from aluminum profiles and medium-density fiberboard panels. These materials provide a stable and reconfigurable laboratory structure while reproducing the relevant geometry of the shuttle interior. The entrance door of the emulator can be opened and closed using push buttons located near the door on both the interior and exterior sides. The emulator is also equipped with an interior lighting system similar to that of the shuttle to reproduce comparable lighting conditions. The system consists of overhead lights above the passenger seats and colored LED light strips installed around the interior of the compartment to provide ambient lighting.

To simulate different daylight conditions, a movable LED matrix is placed outside the emulator. The matrix employs LEDs with a color temperature comparable to natural daylight with high color rendering index and television lighting consistency index values, enabling realistic reproduction of sunlight entering the shuttle cabin through its windows [71,72]. Unlike conventional fixed lighting systems, the position of the LED matrix and its light intensity can be adjusted dynamically, enabling the simulation of various solar illumination conditions, including sunrise, sunset, and different solar angles throughout the day, as well as seasonal differences in sunlight. Furthermore, the matrix provides soft, spatially uniform illumination, thereby minimizing unrealistic shadows and lighting artifacts while enhancing the realism of the mixed reality environment experiments discussed in Section 7.3. These capabilities are particularly important for evaluating lighting conditions that influence human perception, emotional state, and interaction within the simulated environment.

The interior sensing and interaction configuration reproduces the general and individual concepts implemented in the WoZ shuttle. The general configuration monitors and addresses the complete passenger compartment, whereas the individual configuration provides seat-specific monitoring and interaction. The emulator therefore incorporates the same sensor and interface types, technical specifications, mounting positions, and orientations as the corresponding systems in the shuttle. This spatial and technical correspondence allows sensor coverage, passenger visibility, occlusions, interaction concepts, and multimodal data acquisition to be investigated under comparable geometric conditions. It also facilitates the development and preliminary evaluation of passenger monitoring algorithms and HMIs before their implementation in the real shuttle.

A large display is installed at the front of the passenger compartment to provide a visual context corresponding to a shuttle journey. The display has a diagonal of 2.49 m and a resolution of 3840 × 2160 pixels. During an experimental session, the display presents driving scenes that provide participants with a visually plausible impression of traveling in the shuttle. Depending on the investigated scenario, the visual presentation can be supplemented by traffic and vehicle sounds, door opening and closing sounds, acceleration and braking sounds, warning tones, voice announcements, and simulated ambient noise. The combined visual and auditory presentation increases the contextual realism of the simulated journey, although the emulator does not reproduce physical vehicle motion.

The exterior experimental area is restricted to the curbside of the emulator. This side is equipped with the corresponding external sensors and interaction interfaces because the remaining sides are located close to the laboratory walls and do not provide sufficient space for representative external interaction studies. A bus-stop-like structure is positioned on the curbside to reproduce a defined waiting and boarding environment. This arrangement supports investigations of pedestrians and prospective passengers as they approach the shuttle, wait at the bus stop, respond to external information or signals, and prepare to board. It also enables the controlled evaluation of curbside monitoring, pedestrian intention recognition, external HMIs, and communication between the shuttle and waiting passengers. The emulator is therefore not intended to reproduce complete monitoring of the vehicle surroundings but instead provides a defined experimental area for passenger-related curbside interactions.

The emulator uses the same software platform, system architecture, and network configuration as the WoZ shuttle. Consequently, the same or corresponding software components can be used for sensor integration, data transmission, visualization, interaction control, and multimodal data recording. This common technical basis facilitates the transfer of developed software modules, experimental configurations, and data processing procedures between the two platforms. It also allows the sensor and interaction systems to be evaluated in the laboratory before they are deployed in the moving shuttle.

The operator station and the high-performance computing systems are located behind the emulator and remain outside the participants’ field of view during an experimental session. The concealed arrangement prevents the visible presence of the operator from directly influencing the participants’ perception of the automated shuttle scenario. From the operator station, the operator observes the sensor streams and system state in real time, supervises the data recording process, and verifies that the required hardware and software components operate correctly. The operator can also intervene when required because of a technical malfunction, an unexpected participant response, or a safety-related condition. This arrangement combines continuous experimental supervision with the intended perception of an automated and unattended passenger environment.

In contrast to the WoZ shuttle, the emulator does not incorporate propulsion, steering, braking, or other vehicle motion systems and therefore remains stationary in the laboratory. It also does not contain a driver compartment and is not intended for studies of driver behavior, driving performance, or driver-vehicle interaction. Furthermore, it does not reproduce acceleration forces, deceleration forces, cornering, road-induced vibration, or the complete environmental variability of a real journey. The emulator is therefore primarily designed for research on passenger monitoring, human-shuttle interaction, curbside pedestrian interaction, interface evaluation, and the behavioral responses of passengers and prospective passengers.

A principal purpose of the emulator is to support the initial design, implementation, and evaluation of experimental scenarios under controlled conditions. Scenario content, interface behavior, sensor configurations, environmental conditions, and event timing can be standardized across experimental sessions. This control reduces unintended environmental variability and facilitates repeatable and reproducible experiments. Identical scenarios can also be presented to different participants or repeated with modified sensors, algorithms, and interaction concepts, enabling systematic comparisons between experimental conditions. The controlled laboratory environment additionally enables the investigation of simulated unusual or critical situations that are unsafe or operationally impractical during real-world shuttle operation. Examples include unexpected stops, warning situations, communication failures, simulated system malfunctions, or events that require assistance or intervention. Because the emulator remains stationary, such experiments primarily investigate participants’ perceptual, cognitive, emotional, and behavioral responses to the presented situation rather than their physical responses to actual vehicle dynamics.

Findings obtained with the emulator represent the initial controlled stage of the experimental process. At this stage, environmental and operational variability is minimized so that the effects of selected experimental variables can be investigated independently. Subsequent transfer of the scenarios, sensing concepts, and interaction mechanisms to the WoZ shuttle introduces real vehicle motion and additional environmental, traffic-related, and social variability. The comparison between the laboratory and shuttle experiments allows the robustness and transferability of the initial findings to be assessed. It also enables interactions among experimental variables to be examined under progressively more realistic operating conditions. The emulator and the WoZ shuttle therefore form complementary experimental platforms: the emulator provides control and reproducibility, whereas the real shuttle provides increased ecological validity and exposure to real-world variability.

## 6. Use Cases

The proposed emulator mirrors the sensor and interaction capabilities of the automated shuttle, enabling experiments to be conducted under controlled and reproducible conditions before deployment on the real-world platform.

To conduct subject studies using the emulator and shuttle for data collection and analysis, approval from the Ethics Committee of TU Dortmund University was obtained (ID: GEKTUDO_2025-66). At the beginning of each study, participants are informed about the experimental system, the objectives of the study, the data collection and protection procedures, and the overall experimental protocol. They are then asked to provide written informed consent. For studies conducted in the emulator, participants are given the opportunity to familiarize themselves with the system before the actual experiment begins. As the emulator represents an unfamiliar experimental environment for most participants, a short familiarization session, including a preliminary ride in the emulator, is provided prior to data collection.

The following examples draw on previously reported studies to illustrate representative applications of the proposed approach [73,74]. The corresponding findings are summarized here to provide context for the capabilities and potential applications of the proposed approach.

### 6.1. Contactless Monitoring of Passengers’ HR and RR

**Figure 5:**
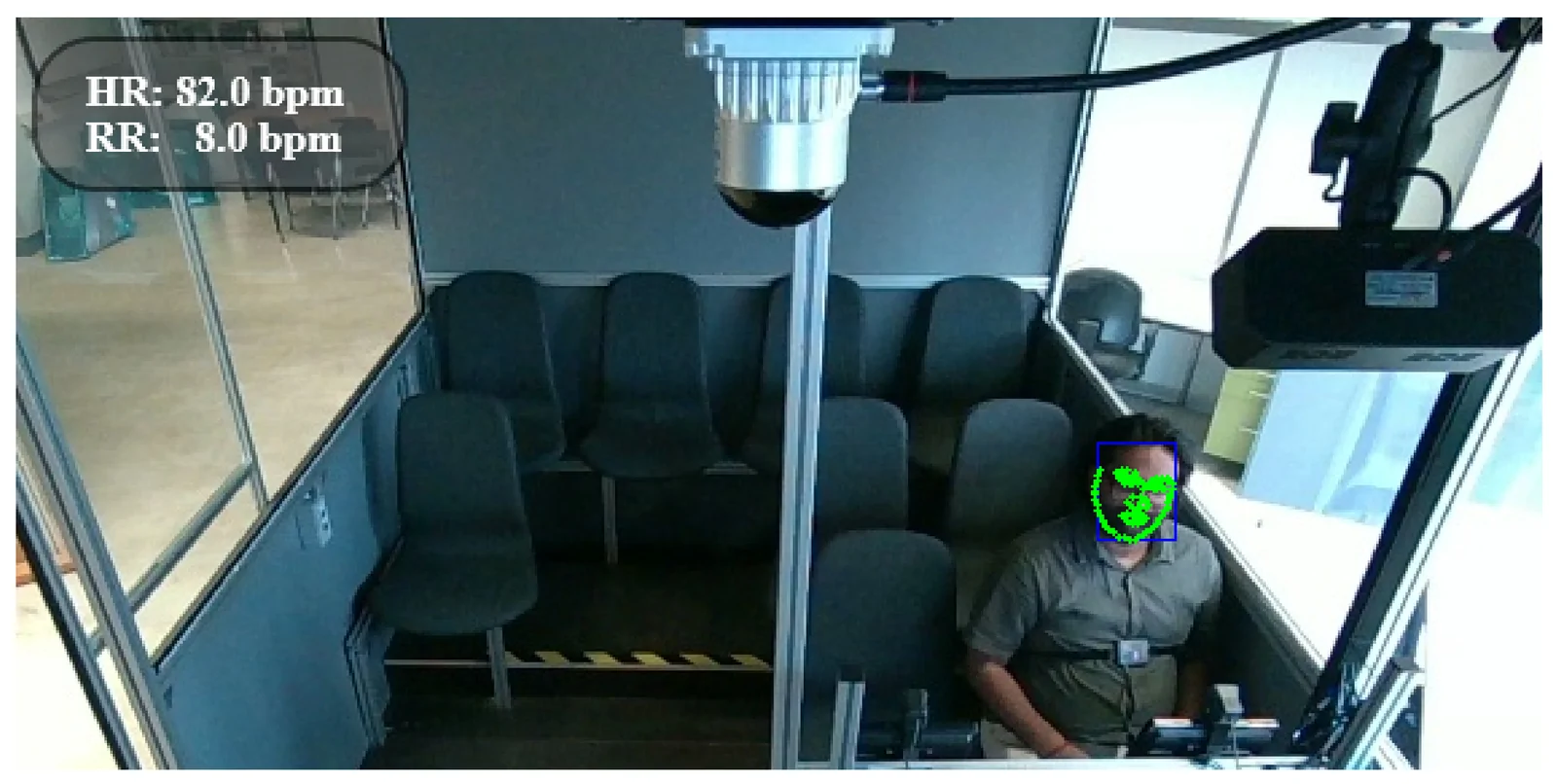
Experimental setup inside the emulator with estimated HR and RR. The blue and green markings represent the detected facial bounding box and landmarks, respectively.

A representative application of the presented experimental system is the non-contact monitoring of physiological vital signs of passengers, specifically HR and RR, without requiring any sensor attachment directly on the body (Figure 5). This exploratory use case demonstrates the system’s capability to serve as a research platform for multimodal physiological sensing under realistic in-vehicle conditions [73].

For this purpose, the experimental system combines sensors based on the following complementary physical measurement principles.

The 24 GHz CW radar detects sub-millimeter displacements of the chest wall induced by both cardiac activity and respiratory excursion. The periodic phase shift of the reflected radar signal encodes the displacement waveform, from which RR and HR are extracted by bandpass filtering in the respective physiological frequency bands followed by spectral peak detection [26,75,76].

The thermal camera exploits the temperature difference between inhaled and exhaled air, which produces periodic temperature oscillations at the nasal aperture that are detectable as an RR signal. Additionally, the pulsatile blood flow in superficial facial vessels induces cyclic skin temperature changes in the millikelvin range, providing a thermal HR signal [68].

Three RGB-D cameras and a webcam contribute two further measurement modalities: motion analysis and remote photoplethysmography (rPPG). In the motion-based approach, subtle vertical displacements of the head and thorax, driven by the cardiac ballistic force and respiratory chest expansion, respectively, are tracked via optical flow algorithms applied to facial and upper-body landmarks [77,78]. The depth channel of the cameras additionally enables direct reconstruction of the 3D thorax displacement, providing an optical analog to the radar measurement [79]. The rPPG approach exploits the color change in facial skin caused by the cyclic variation in blood volume beneath the surface, extracting the cardiac pulse from the intensity signal of the RGB channels averaged over the facial region of interest [80].

For this exploratory evaluation, four participants completed six experimental scenarios, each lasting five minutes, involving predefined sitting positions, movement patterns, and clothing conditions to assess the robustness of the individual sensor pipelines and the sensor fusion. Reference HR was obtained from the ECG integrated into the radar sensor, while RR was measured using the respiratory belt. Each sensor pipeline synchronously produced updated HR and RR estimates every two seconds, with rate estimation based on a 60-s analysis window. The individual sensor estimates are combined in a decision-level sensor fusion [81], using signal-to-noise-ratio-weighted averaging [82], with the weights determined from the spectral quality of the respective modality at each analysis window. The evaluation was intended to demonstrate the feasibility of the multimodal setup rather than to provide a statistically powered validation of contactless vital-sign estimation.

Across all modalities, signal extraction follows a unified processing backbone of interpolation, bandpass filtering, Hanning-windowed spectral analysis, and dominant frequency identification.

In the calm scenario, participants are not instructed to perform any specific activity, and they remain seated in their normal positions. Under these conditions, the sensor fusion approach achieves a maximum mean absolute error (MAE) of 0.88 bpm and a correlation coefficient of 0.84 for RR. The more subtle heart movements lead to an MAE of 2.11 bpm with a correlation coefficient of 0.84 for HR. These results illustrate the potential of the experimental platform for multimodal contactless vital-sign monitoring, while broader validation across participants, conditions, and recording sessions remains subject to future work.

### 6.2. Investigating Social Influence on Affective and Behavioral States in Critical Scenarios

The first use case focused on each passenger individually. However, since the shuttle represents a shared public space, investigating scenarios involving multiple passengers is also essential. In the absence of a bus driver, the social dynamics within the shuttle may change substantially. Therefore, this use case investigates the influence of social context on passengers’ affective and behavioral states [74].

The study focuses on passengers’ experiences and passive social influence between passengers across three scenarios in the emulator: (i) a baselinescenario without a social component; (ii) a social scenario including a social component under urban traffic conditions; and (iii) an alone scenario without a social component under rural traffic conditions. The scenarios are implemented using a display presenting prerecorded driving scenarios and speakers reproducing driving-related and critical-situation sounds. The chosen driving scenarios are comparable in weather, time of day, traffic density and the distribution of different road users, reducing the influence of environmental factors on the participants’ affective and behavioral responses. The social component is implemented by including confederate passengers, who are instructed to behave passively and neutrally, without interacting with the participant. The social component is designed to investigate whether the presence of other passengers influences the participant’s affective and behavioral responses to critical events during the emulated shuttle rides.

A total of 16 participants (8 male and 8 female) are included in this study. A stratified 2×2 experimental design is employed to counterbalance the order of the two scenarios across male and female participants. Both subjective and objective data are collected. To assess passengers’ instantaneous affective states following each critical event occurring during the shuttle ride, the Self-Assessment Manikin (SAM) is administered. Before the scenarios, demographic information is collected, including age, prior knowledge of automation technology, and frequency of public transport use. In addition, following the baseline scenario, two questionnaires, Social Interaction Anxiety Scale (SIAS) and Extended Technology Acceptance Model (ETAM), are administered to assess social anxiety and acceptance- and usability-related perceptions of the emulator (as automated shuttle), respectively. The proposed approach extends the framework introduced by Davis [83] by incorporating perceived usefulness, perceived ease of use (PEOU), behavioral intention, trust, perceived risk, perceived comfort, and attitude toward automated buses, thereby enabling a more comprehensive assessment of passengers’ perceptions and affective experiences. Between the subsequent two scenarios, the Positive and Negative Affect Schedule (PANAS) rating is administered to obtain retrospective affective responses, complementing the instantaneous SAM ratings.

The results demonstrate systematic changes in passengers’ self-reported affective states in response to critical events (Table 5). The findings further indicate that passengers’ affective experiences in automated shuttles are influenced by the nature of the critical event and the social context, as well as by their acceptance-related perceptions of the technology.

In parallel with the subjective measures, objective behavioral data are collected using RGB-D cameras to capture facial reactions as potential behavioral markers of passengers’ affective states. For each time step, probabilities are obtained for eight facial expression classes: neutral, happiness, sadness, surprise, fear, disgust, anger, and contempt. The influence of the social context on participants’ facial expressions is investigated using linear mixed-effects models for the standard deviation of the emotion probabilities for fear, sadness and surprise. The social context and PEOU are found to be significant positive predictors for all fitted variability-based emotion features. It is argued that the presence of other participants may increase self-monitoring and expressive regulation, while the PEOU may reflect a socially adaptive affect regulation strategy. It should be noted that participants travel alone in the rural scenarios, whereas social rides are conducted exclusively in urban scenarios. Consequently, the traffic environment represents a potential confounding variable that should be considered when interpreting the results.

Additional qualitative insights into the perceived realism and automatedness of the emulator are obtained from participants’ free-text feedback. One participant described the simulated environment as highly realistic, particularly the boarding of additional passengers at bus stops. Another noted that, during public transport journeys, the distinction between human-operated and automated driving becomes less relevant, as attention is primarily directed toward the journey itself. Several participants expressed concerns about simulated vehicle behavior, including sharp cornering, unusual mechanical noises, and apparent technical malfunctions, suggesting that the presented scenarios elicited responses associated with real vehicle operation. However, one participant questioned whether the simulated experience would correspond to an actual shuttle journey. Overall, these subjective observations provide preliminary indications of the emulator’s realism and its ability to engage participants in the simulated journey, although perceived automatedness is not directly measured.

## 7. Discussion and Conclusions

This contribution presents an integrated experimental platform for human-centered research on future automated public transport. The platform combines a moving nine-seat WoZ shuttle, a full-scale laboratory emulator (analog twin), multimodal interior and exterior sensing, HMIs, and a modular ROS 2-based architecture for synchronized data acquisition. Its main contribution is a technically transferable experimental infrastructure for developing and evaluating passenger monitoring, environmental perception, assistance, and interaction functions rather than the verification and validation of a single algorithm.

A central feature of the concept is the separation between simulated and technically implemented automation. A concealed human driver performs the dynamic driving task and ensures operational safety, while passenger monitoring, environmental perception, event detection, and interaction functions are implemented through sensors, algorithms, and interfaces. This allocation enables non-driving functions to be investigated without requiring a complete automated driving system and prevents the operator from replacing the algorithms under evaluation.

### 7.1. Experimental Platform and Transferability

Previous studies have investigated automated shuttles with visible or hidden safety operators [2,5,7,8,9] or have used a concealed driver to simulate a robo-taxi service [6]. The present platform extends these approaches to a shared public transport environment with multiple passengers, whole-compartment and seat-specific monitoring, boarding-related studies, and communication with surrounding road users. Compared with the static shuttle mock-up presented by Escher et al. [11], the laboratory emulator reproduces the relevant geometry, sensors, interfaces, and software configuration of the moving shuttle. This correspondence supports the transfer of software modules between controlled laboratory studies and real journeys.

The complementary use of the emulator and the WoZ shuttle provides both experimental control and ecological validity. The emulator permits standardized scenario timing, environmental conditions, interface behavior, and the safe investigation of unusual or critical situations. The moving WoZ shuttle introduces vehicle motion, vibration, traffic, ambient noise, and social variability. Conducting corresponding experiments on both platforms can therefore reveal whether findings obtained under controlled conditions remain valid under real operating conditions. The use cases presented in this work are based on previously reported studies and illustrate representative applications of the platform.

### 7.2. Limitations and Future Experimental Work

Several limitations must be considered. The emulator platform does not reproduce the behavior of an automated driving system. Human driving may differ in trajectory planning, acceleration, braking, gap acceptance, and responses to ambiguous traffic situations. The platform is therefore unsuitable for assessing automated driving performance or safety. The emulator does not reproduce inertial cues, road-induced vibration, or the complete external environment. Individual modalities are also affected by operating conditions, including illumination, occlusion, passenger motion, temperature, and vehicle vibration. Finally, results obtained with one vehicle configuration, route, or participant population may not generalize directly to other shuttle designs or user groups. The generalizability of findings obtained using the proposed platform is limited by the shuttle model, participant population, and route characteristics. The current implementation uses an eVersum mobility electro shuttle MK II-400 with nine passenger seats and a corresponding laboratory emulator. Its specific cabin geometry, seating arrangement, sensor positions, and interaction interfaces may influence sensing performance, passenger behavior, and the perception of the simulated automated journey. Consequently, transferring algorithms or interaction concepts to other shuttle models may require modifications to the experimental configuration and renewed validation. Generalization across passenger populations also remains to be established, as the illustrative use cases presented here do not demonstrate representativeness of the wider public transport population. Findings should therefore be interpreted in relation to the characteristics of the participants studied, including age, prior experience with automation, and frequency of public transport use. Their applicability to user groups with different needs, including older adults and passengers with mobility or sensory impairments, requires further investigation. Regarding route characteristics, operation of the real shuttle along a predefined route and the presentation of selected urban and rural driving scenarios in the emulator do not establish generalizability across the full range of public transport environments. Differences in traffic density, operating speed, stop frequency, road conditions, and passenger occupancy may affect both measurement quality and passengers’ perceived safety, trust, and interaction behavior. Thus, although the modular architecture supports technical adaptation, broader generalizability of the experimental findings requires comparative studies involving additional shuttle configurations, diverse participant groups, and multiple route types and service patterns, including demand-responsive operation.

In the next steps, equivalent subject studies will be conducted in the laboratory emulator and the WoZ shuttle using similar scenarios, protocols, sensor configurations, and outcome measures. Direct comparison of the results can quantify platform-induced differences and determine the extent to which findings obtained under controlled laboratory conditions transfer to real shuttle journeys. This approach provides a basis for progressively increasing the complexity and realism of experimental scenarios while maintaining control over their design and execution.

### 7.3. Virtual and Mixed Reality as a Future Research Direction

Building on this experimental foundation, a further direction for future research is the integration of virtual and mixed reality (VR/MR) into the existing platform. Such an extension could enable the controlled and reproducible investigation of critical situations that are difficult to recreate safely and systematically in real-world shuttle studies. Incidents in public transport can differ considerably in their type, severity, duration, and social context, while their systematic investigation is important because such events may influence passengers’ trust in automated shuttles and remain comparatively understudied [84,85]. VR/MR could provide a means of varying these characteristics while maintaining control over the experimental conditions. Potential scenarios include technical failures, medical emergencies, threatening situations, and other unexpected events.

An additional research direction concerns the comparison of different forms of immersive simulation. Shin et al. [86], for example, reported a higher sense of plausibility illusion in VR than in MR, contrary to the initial expectation. Future experiments could investigate whether similar differences arise in automated shuttle contexts and whether characteristics of the physical experimental environment, such as the controlled lighting conditions and LED-based matrices, influence the perceived plausibility of the simulated scenario. Compared with existing studies employing VR in automated shuttle contexts, such as the work of Wu et al. [87], the proposed extension would place particular emphasis on the reproducible simulation of critical incidents within a physical shuttle environment.

The combination of MR with the physical components of the laboratory environment could further increase the realism of such investigations. The shuttle emulator, selected passengers or other persons, and physical elements of a bus-stop environment could be combined with virtual representations of the surrounding road environment, other road users, virtual characters, and dynamic events. This approach would allow real and virtual elements to be integrated while preserving the reproducibility of the experimental scenario.

The existing sensing infrastructure could be used to investigate passenger responses throughout these scenarios. RGB-D cameras, for example, could support the observation of body movements, facial expressions, and other behavioral cues [88,89]. Such data could be used to estimate passengers’ affective and emotional states and to investigate changes in behavioral responses during critical situations. At the same time, MR glasses could provide additional behavioral measures, including eye movements, gaze direction, and pupil-related measures [90,91]. These measures could be used to investigate the behavioral state of the person wearing the glasses and to evaluate the effectiveness of different HMI interventions. For example, gaze data could provide information on the time required for a passenger to notice and attend to an HMI notification, while subsequent behavioral responses could indicate whether and how the passenger reacts to the presented information. The combination of such measures with contextual information about the simulated situation could therefore support a more comprehensive investigation of passengers’ behavioral and affective states.

An interactive VR/MR environment could also extend the investigation beyond passive observation. Participants could actively interact with the simulated environment and influence the progression of a scenario through their decisions and actions. This would provide an opportunity to investigate decision-making and active behavioral responses under controlled but realistic conditions. Such an approach has also been explored in interactive virtual environments, where participant actions can influence the course of a simulated situation and provide behavioral measures beyond passive observation [92]. In the context of automated public transport, these capabilities could support the development of more adaptive HMIs that consider both the passenger’s current state and the evolving context of an event [93].

Finally, the combination of VR/MR with the existing emulator and WoZ shuttle provides a potential pathway from controlled simulation to in-field validation. Since the emulator incorporates the same sensing and functional components as the real shuttle, scenarios developed in the virtual or mixed-reality environment could subsequently be transferred to the real vehicle for selected validation studies [94]. Comparing responses across these stages could provide insight into how passenger behavior and affective responses change with increasing environmental and physical realism. In this way, critical incidents could first be developed and systematically investigated under controlled conditions and subsequently reproduced in the real shuttle to examine the transferability and ecological validity of the findings.

Overall, the proposed platform provides a transferable experimental foundation for human-centered research in automated public transport. Its modular structure and combination of controlled laboratory experimentation with realistic shuttle operation offer a basis for progressively developing, evaluating, and validating future passenger-oriented technologies under increasingly realistic conditions.

## Figures and Tables

**Table 1:** Factors influencing passengers’ perception and acceptance of automated shuttles.

Factor	Description	Reference
Shuttle speed	Lower speed increases perceived safety.	[3,7,9]
Driving behavior	Driving characteristics such as smooth braking and cornering facilitate sensed safety.	[7,9]
Operating environment	Controlled environments like rural areas are preferred over urban traffic areas.	[9]
Trust in the technology	Previous accidents and perceived technical limitations lead to concerns.	[3,4,8,9]
Interaction between shuttle and passengers	Human-machine interface (HMI), information provision, and signaling influence passengers’ perceived transparency and trust.	[5,6,9]
Absence of an onboard operator	Concerns related to the lack of a human operator who can intervene in unexpected situations.	[3,5,7,8]
Dynamic object recognition and event detection	The ability of the shuttle to detect and respond to dynamic objects and unexpected events.	[9]

**Table 2:** Comparison of existing automated-shuttle research platforms and the experimental platform presented in this work. GNSS: Global Navigation Satellite System; ECG: Electrocardiography; HR: Heart Rate; RR: Respiration Rate.

Study	Hidden Driver	Moving Vehicle	Lab Twin	Interior Sensing	Physio. Sensing	External Perception	HMI	Sync. Log.	Lab-Field Transfer
[2]	✓ (safety controller)	✓	–	–	–	–	–	–	–
[9]	✓ (hidden steward)	✓	–	–	–	Lidar, geo-positioning	✓	–	–
[6]		✓ (car)	–	Video camera	–	–	–	–	–
[5]	✓ (safety instructor)	✓	–	–	–	–	✓	–	–
[7]	✓ (safety operator)	✓	–	RGB	–	RGB	✓	–	–
[8]	✓ (safety operator)	✓	–	Muse 2	EEG	Lidars, camera, GNSS, odometry	–	✓	✓
[11]	–	–	✓	–	–	–	✓	–	–
This work	✓	✓	✓	RGB-D, RGB, Thermal, Radar, Lidar, ECG, Respiration Belt	HR, RR	RGB, Thermal, Lidar	✓	✓	✓

✓: yes, –: no.

**Table 3:** Grouped sensor latency before and during MCAP recording. Latencies are reported as message age in milliseconds. The LiDAR values refer to the Autoware PublishedTime publication age of the cropped point cloud.

Sensor Group	Rate [Hz]	Median [ms]	p95 [ms]
Blackfly RGB	21.0→20.9	5.1→10.6	11.5→29.1
RealSense RGB	26.2→21.8	176.7→178.7	234.8→321.8
RealSense aligned depth	20.4→11.2	186.0→194.2	232.6→325.5
Logitech RGB	15.7→18.3	43.7→51.2	52.6→76.2
FLIR A70 LWIR	9.1→10.9	119.3→196.1	168.9→293.2
LiDAR	20.0→20.0	1.0→3.2	3.2→13.2

**Table 4:** Ping-pong latency before and during MCAP recording. The one-way latency is approximated as half of the round-trip time (RTT) after subtracting the responder processing time. The ping-pong test confirms that recording adds general communication/runtime delay.

Metric	Before MCAP [ms]	During MCAP [ms]	Change [ms]
Median RTT	3.9	8.5	+4.6
p95 RTT	7.1	21.4	+14.3
p99 RTT	61.0	128.0	+67.0
Maximum RTT	113.6	235.2	+121.5
Median one-way estimate	1.9	4.2	+2.3
p95 one-way estimate	3.5	10.7	+7.2

**Table 5:** Results of Ordinal Logistic Regression between PAD-mapped SAM ratings and fixed effects. Fixed-effect coefficients with standard errors (SE) β±SE and *p*-values are reported. Taken from [74].

Fixed Effect	Pleasure	Arousal	Dominance
Condition	−2.031 * ± 0.42	1.664 * ± 0.42	−1.209 * ± 0.46
	<0.01	<0.01	<0.01
SP	0.926 * ± 0.40	−0.371 ± 0.38	−0.03 ± 0.31
	0.02	0.33	0.92
Trust in Automated Shuttles	7.143 * ± 1.26	-	-
	<0.01	-	-

(*) denotes p<0.05.

## Data Availability

The platform performance measurements presented in this study are available on request from the corresponding author.

## Abbreviations

The following abbreviations are used in this manuscript:

CAN	Controller Area Network
CW	Continuous wave
ECG	Electrocardiography
EEG	Electroencephalography
ETAM	Extended Technology Acceptance Model
HMI	Human-machine interface
HR	Heart rate
LWIR	Long-wave infrared
MAE	Mean absolute error
MR	Mixed reality
OS	Operating system
PANAS	Positive and Negative Affect Schedule
PAULA	Platform for automated user-centred local mobility analysis
PEOU	Perceived ease of use
ROS	Robot Operating System
rPPG	Remote photoplethysmography
RR	Respiratory rate
SAM	Self-Assessment Manikin
SDK	Software Development Kit
SP	Social presence
SIAS	Social Interaction Anxiety Scale
VR	Virtual reality
WoZ	Wizard-of-Oz
